# Comparison of Pressure-Retarded Osmosis Performance between Pilot-Scale Cellulose Triacetate Hollow-Fiber and Polyamide Spiral-Wound Membrane Modules

**DOI:** 10.3390/membranes11030177

**Published:** 2021-02-28

**Authors:** Yuriko Kakihana, Nora Jullok, Masafumi Shibuya, Yuki Ikebe, Mitsuru Higa

**Affiliations:** 1 Graduate School of Sciences and Technology for Innovation, Yamaguchi University, 1677-1 Yoshida, Yamaguchi 753-8511, Japan; kakihana@yamaguchi-u.ac.jp (Y.K.); glory.color413@gmail.com (M.S.); a066vfu@yamaguchi-u.ac.jp (Y.I.); 2 Blue Energy Center for SGE Technology (BEST), 2-16-1 Tokiwadai, Ube, Yamaguchi 755-8611, Japan; 3 Faculty of Chemical Engineering Technology, Universiti Malaysia Perlis, Kompleks Pusat Pengajian Jejawi 3, Jejawi 02600, Perlis, Malaysia; norajullok@unimap.edu.my; 4 Centre of Excellence for Biomass Utilization, Universiti Malaysia Perlis, Pusat Pengajian Jejawi 3, Jejawi 02600, Perlis, Malaysia

**Keywords:** pressure-retarded osmosis, pilot scale, hollow fiber, spiral wound, power density

## Abstract

Pressure-retarded osmosis (PRO) has recently received attention because of its ability to generate power via an osmotic pressure gradient between two solutions with different salinities: high- and low-salinity water sources. In this study, PRO performance, using the two pilot-scale PRO membrane modules with different configurations—five-inch cellulose triacetate hollow-fiber membrane module (CTA-HF) and eight-inch polyamide spiral-wound membrane modules (PA-SW)—was evaluated by changing the draw solution (DS) concentration, applied hydrostatic pressure difference, and the flow rates of DS and feed solution (FS), to obtain the optimum operating conditions in PRO configuration. The maximum power density per unit membrane area of PA-SW at 0.6 M NaCl was 1.40 W/m^2^ and 2.03-fold higher than that of CTA-HF, due to the higher water permeability coefficient of PA-SW. In contrast, the maximum power density per unit volume of CTA-SW at 0.6 M NaCl was 4.67 kW/m^3^ and 6.87-fold higher than that of PA-SW. The value of CTA-HF increased to 13.61 kW/m^3^ at 1.2 M NaCl and was 12.0-fold higher than that of PA-SW because of the higher packing density of CTA-HF.

## 1. Introduction

Energy demand is increasing globally, in tandem with economic development and population growth. Because the majority of primary energy sources comes from fossil fuels [1], the world is facing crucial challenges in meeting energy-source demands, owing to the decrease in the accessibility of cheap fossil fuels [2]. Moreover, damaging greenhouse gas emissions ultimately contribute to climate change [3]. Many research efforts have focused on developing efficient alternative energy sources: Solar, wind, tidal, wave, and biomass have been extensively studied as alternative and sustainable energy sources. However, the varying availability of energy sources, complex logistics, and high installation costs still prevent their widespread implementation.

Furthermore, water shortages are among society’s most challenging problems, for which seawater reverse-osmosis desalination (SWRO) is a promising solution. Although the concentrated brine coming from desalination plants sometimes causes environmental problems [4], owing to its high salinity, it can be used as a valuable energy resource referred to as salinity gradient energy (SGE) [5,6,7,8]. SGE is a renewable energy source that is obtained by mixing two salt solutions with different salinities [9,10]. There are two main membrane-based technologies that convert SGE into electricity: reverse electrodialysis (RED) [9,11,12] and pressure-retarded osmosis (PRO) [10,13]. RED generates energy by converting counter-ion permselective transport with ion-exchange membranes to electric current by redox reactions at two electrodes; there have been reports on large-scale RED systems [14,15,16,17]. In contrast, in a PRO system, a semi-permeable membrane (SPM) separates a low-salinity solution, the feed solution (FS), from a pressurized high-salinity solution, called the draw solution (DS).

The advantages of using SWRO brine as the DS of PRO or RED are as follows: The high salinity of the brine provides high power output for RED and PRO systems and the intake, and the pretreatment of DS supplied to the two systems is associated with a lower cost, as well as increased energy savings.

Both the technologies have associated advantages, as well as disadvantages: The gross power density of PRO is higher than that of RED; however, especially in small-scale plants, the low energy conversion efficiency of the high-pressure pumps, pressure exchanger, turbine, and generator of PRO results in a lower net power than in the RED. The effect of the bivalent ions in the seawater and the SWRO brine increases the power output of PRO, while decreasing the output of RED. Currently, the cost of membranes for RED is higher than that for PRO [18].

There was an attempt to compare both PRO and RED, using experimental data obtained from the literature; however, it was unsuccessful, owing to incomplete sets of equal performance data. In 2007, Post et al. [5] developed a model in which each equation was compared to its equivalent for PRO and RED. Based on the developed model, both with respect to power density and energy recovery, it was concluded that each technique has its own field of application; PRO is more suitable for power generation using concentrated saline brines, whereas RED is more effective for power generation using seawater and river water. Details of the comparison between PRO and RED based on the developed model can be found in the literature [5].

**Figure 1:**
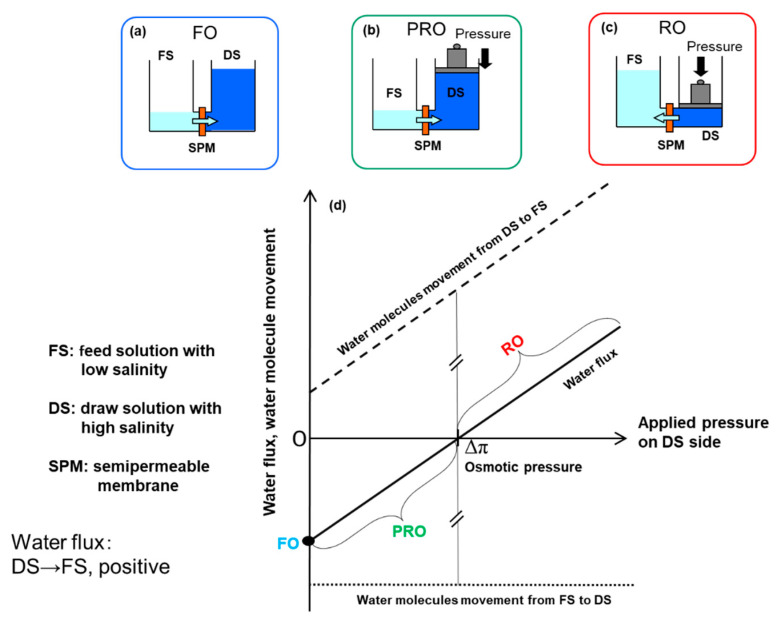
Water flux and water molecule movement in a dialysis system consisting of semi-permeable membrane (SPM), feed solution (FS), and draw solution (DS), to explain principle of forward osmosis (FO), pressure-retarded osmosis PRO, and reverse osmosis (RO).

In PRO, the osmotic water flow occurs from the FS to the DS through the membrane and propels a turbine connected to a generator to generate electricity. Figure 1 shows the water flux and water molecule movement in a dialysis system consisting of SPM, FS, and DS, which explains the principle of forward osmosis (FO), PRO, and reverse osmosis (RO). Here, SPM is assumed to have ideal water permeability. First, there is no hydrostatic pressure difference between the FS and DS sides. In this case, the movement of water molecules from FS to DS (WM1), shown by the dotted line in Figure 1, is higher than that from DS to FS (WM2), shown by a broken line, because the thermal mobility of water molecules near the membrane surface at the FS side is higher than that at the DS side because of the presence of non-permeable ions in DS. Therefore, the net water flux defined by subtracting WM1 from WM2 shows a negative value, as shown by the solid line in Figure 1. This phenomenon is called forward osmosis. When the hydrostatic pressure difference (Δ*P*) is applied to the DS side, WM2 increases with increasing Δ*P*, while WM1 is independent of the pressure. Hence, the net water flux increases with increasing Δ*P*. At the point where WM1 is equal to WM2, the net water flux becomes zero. The Δ*P* at this point is called the osmotic pressure difference (Δ*π*) between the DS and FS. In the case where Δ*P* is larger than Δ*π*, the net water flux becomes a positive value, which is called the reverse osmosis mode. PRO is operated at applied hydrostatic pressures 0 < Δ*P* < Δ*π* so that the volume of pressurized DS increases by the net water flux to rotate a turbine to generate energy [19].

Realizing the potential of PRO, intensive studies have been conducted, using laboratory-scale PRO analysis, utilizing coupon-size membranes [20,21,22,23,24,25,26] and small hollow-fiber (HF) modules [27,28,29,30,31,32,33]. Several bench-scale investigations have demonstrated promising results and, to some extent, the membrane power density reaches 60 W/m^2^ at a high pressure of 48 bar with a 3 M NaCl DS using a thin-film composite membrane [34]. However, with regard to upscaling, the performance of the scaled-up prototype may differ significantly from the laboratory data. This is because the real plant scale of the PRO system, for instance, depends not only on the membrane parameters but also on the membrane orientation/membrane configuration, and the operational conditions such as the flow rates of the DS and FS and the operating pressure [10,35]. The need for the evaluation of power generation utilizing PRO at the near industrial scale is therefore vital to determine its real potential and to narrow down the gap between theoretical and practical applications. To understand the effect of the abovementioned factors on PRO performance, evaluations using spiral-wound (SW) modules [36,37] and HF modules were investigated. Saito et al. [4] performed PRO tests of a 10-inch HF module using high-concentration brine from a seawater RO plant and sewage-treated water from a regional sewage-treated center as DS and FS, respectively, and obtained 7.7 W/m^2^ of a maximum gross power density at 2.5 MPa of hydraulic pressure difference. Sakai et al. [38] obtained 13.5 W/m^2^ of power density, using a 10-inch HF PRO module. Experiments and simulation studies of PRO performance, using pilot-scale PRO HF modules under various operational conditions, have also been performed [35,39,40,41,42,43,44,45,46]. In previous studies [35,43], the module performance with volumetric-based power outputs, as well as membrane-area-based power density, was investigated, to compare the PRO performance between the two different module configurations. However, to the best of the authors’ knowledge, there have been no studies on systematic PRO performance evaluation of pilot-scale SW and HF membrane modules based on experimental data under the same operating conditions, to compare the PRO performance between SW and HF configurations. In this study, the performance of two types of pilot-scale membrane modules with HF and SW configurations is investigated under a wide range of PRO operating conditions: DS concentrations, the flow rates of DS and FS, and applied hydraulic pressure differences; furthermore, the PRO performance between the two modules is compared to provide useful insights into the design of large-scale PRO plants.

## 2. Experimental

### 2.1. Materials and Chemicals

Tap water was first treated with activated carbon to remove any traces of chlorine prior to use as the FS and for the preparation of DS. DS was prepared by using analytical-grade sodium chloride (NaCl, Nacalai Tesque Co., Ltd., Kyoto, Japan) dissolved in chlorine-free tap water.

### 2.2. Cellulose Triacetate Hollow-Fiber (CTA-HF) and Polyamide Spiral-Wound (PA-SW) Membrane Modules

An open-ended HF type of cellulose triacetate (CTA) designed for PRO applications from Toyobo Co. Ltd (CTA-HF) and a prototype polyamide thin film composite membrane module (PA-SW) were used. The membrane modules have four open ports: an inlet and an outlet for DS and FS. The specifications of CTA-HF and PA-SW are listed in Table 1.

### 2.3. RO Experiment

**Figure 2:**
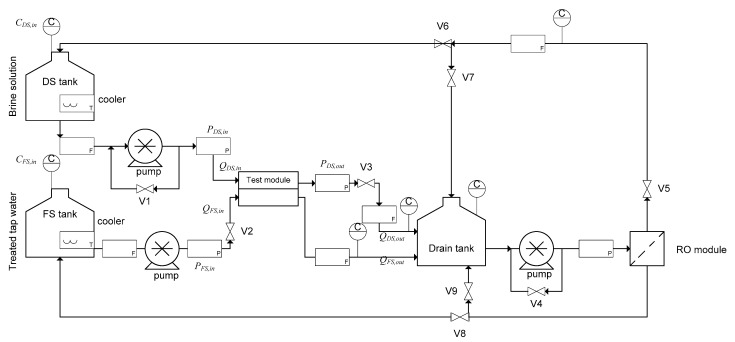
Schematic of the pilot-scale PRO evaluation system used in this study. C represents conductivity meter, F is flow meter, V is valve, and P is the pressure gauge.

A preliminary analysis in reverse osmosis (RO) mode was conducted to determine the water permeability (*A*) and salt permeability (*B*) of the membrane used in the two CTA-HF and PA-SW modules. Figure 2 presents a schematic of the evaluation system of a pilot-scale PRO module. The system was the same as in a previous study [35], and was also used for the RO evaluation test in this study. The concentration of FS in the test was 500 ppm NaCl. To operate the system in RO mode, valve V2 was completely closed, so that the permeated water flowed out from the outlet at the FS side of the module. The activated carbon-treated tap water was fed into the DS side of the membrane module with an inlet flow rate of 4.0 L/min. The applied pressure difference between the DS and FS was carefully controlled, using valve V3. The water permeation flux was measured, using a flow meter at the FS outlet. The *A* (m s^−1^ Pa^−1^) value was then determined from the slope of the graph plotted for water flux over the applied pressure according to Equation (1):

*J_w_* = *A* (Δ*P* − Δ*π*) (1)

where *J_w_* is the water flux (L m^−2^ h^−1^), Δ*P* is the hydraulic pressure difference, and Δ*π* is the osmotic pressure difference. To determine the value of *B*, Equation (2) was applied [25]:

(2) $$B=\frac{A\left(\Delta P-\Delta \pi \right)\left(1-R\right)}{R}$$

where the rejection rate, *R*, is calculated, using Equation (3).

(3) $$R=1-\frac{C_{p}}{C_{F}}$$

where *C_p_* is the concentration at the permeate side, and *C_F_* is the concentration at the feed solution.

### 2.4. PRO Experiment

To evaluate the performance in PRO mode, a PRO test was performed, using the system shown in Figure 2. Tap water was fed to the system as FS, using a low-pressure pump, and NaCl solutions of various concentrations were fed as DS. The inlet concentrations of FS and DS were measured, using a conductivity meter; the flow rates of the inlet and outlet at the DS and FS sides were measured, using flow meters; and the pressure at each position (*P*_FS,in_, *P*_DS,in_ and *P*_DS,out_) was measured, using a pressure gauge. The inlet flow rate of the FS was set between 2.0 and 6.0 L/min, while the DS inlet flow rate was between 2.0 and 10.0 L/min. The maximum flow rates of FS and DS at the tests were 6.0 and 10 L/min, respectively, owing to the pump limitation created by the evaluation system.

During PRO evaluation, DS was diluted by the permeated water from the FS, and the salt concentration in the FS was increased by the reverse salt flux from the DS (DS leakage). A regeneration system with RO was added after the PRO unit to obtain steady-state conditions during the PRO test. To regenerate both the DS and FS, the needle valves V4 and V5 were used to control the concentration of the brine and permeate at their respective initial FS and DS concentrations. Thus, a steady-state long-standing module operation was achieved, using a hybrid PRO and RO regeneration unit. Each data point was logged and recorded on a computer 5 min after the parameter was adjusted at 10 s intervals. The PRO performances of the CTA-HF and PA-SW membrane modules were evaluated under various operating parameters. The permeate water flow rate (*Q_w_*) through the module was determined, using the inlet and outlet flow rates of FS, $Q_{\mathrm{FS},\mathrm{in}}\;\mathrm{and}\;Q_{\mathrm{FS},\mathrm{out}}$, respectively, using Equation (4):

(4) $$Q_{w}=Q_{\mathrm{FS},\mathrm{in}}-Q_{\mathrm{FS},\mathrm{out}}$$

The permeate flux (*J_w_*) through the membrane module was determined, using Equation (5), and is the division of the permeate water flow rate by the membrane area (*S_m_*).

(5) $$J_{w}=\frac{Q_{w}}{S_{m}}$$

The permeation rate (*η*) of the FS side was determined by using Equation (6):

(6) $$\eta \equiv \frac{Q_{w}}{Q_{\mathrm{FS},\mathrm{in}}}\times 100\%$$

When *η* = 100, all solutions on the FS side permeated to the DS side. The dilution factor (*φ*) on the DS side was defined, using the inlet flow rate of the DS ($Q_{\mathrm{DS},\mathrm{in}}$) and *Q_w_*, as in Equation (7):

(7) $$\varphi \equiv 1+\frac{Q_{w}}{Q_{\mathrm{DS},\mathrm{in}}}$$

When *Q*_DS,in_ » *Q_w_*, *φ* is close to unity. This implies that the DS outlet concentration was nearly equal to the DS inlet concentration, indicating that the DS dilution effect was negligible.

To estimate the reverse salt flux (*J_s_*), Equation (8) was employed:

(8) $$J_{s}=\frac{\left(Q_{\mathrm{FS},\mathrm{out}}\times C_{\mathrm{FS},\mathrm{out}}-Q_{\mathrm{FS},\mathrm{in}}\times C_{\mathrm{FS},\mathrm{in}}\right)}{S_{m}}$$

Many PRO evaluations in the literature [10,12,13,19,20,21,22,23,24,25,26,27,28,29,30,31,32,33,34] are based on the power density per membrane area to characterize the membrane performance. The membrane area-based power density (*PD*^area^) is defined as the effective membrane area-based power density and is calculated by using Equation (9):

(9) $$PD^{\mathrm{area}}=J_{w}\Delta P$$

From an industrial point of view, module volume-based PRO performance is vital because the volume-based size and number of required modules are necessary for designing a full-scale PRO plant [35,43,45]. The module volume-based gross power density ($PD^{\mathrm{vol}}$) used in this study is expressed as follows:

(10) $$PD^{\mathrm{vol}}=J_{w}^{\mathrm{vol}}\Delta P$$

$J_{w}^{\mathrm{vol}}$ is module volume-based water flux expressed as

(11) $$J_{w}^{\mathrm{vol}}=\;\frac{Q_{w}}{V_{\mathrm{mod}}}$$

where *V*_mod_ is the module volume. From (9) and (10), $PD^{\mathrm{vol}}$ is expressed, using *PD*^area^ as follows:

(12) $$PD^{\mathrm{vol}}=\beta PD^{\mathrm{area}}$$

where *β* is the packing density of the membrane module and is defined by using the following equation:

(13) $$\beta =S_{m}/V_{\mathrm{mod}}$$

The power extracted from the PRO, using SGE, was determined by calculating the conversion efficiency to electrical energy. Electrical energy can significantly contribute to the establishment of the optimum operating conditions. To obtain the optimum operating conditions, the generated net power output (*W*_net_) must first be determined, using Equation (14) [38]:

(14) $$W_{\mathrm{net}}=W_{\mathrm{gross}}-\left(CE_{\mathrm{DS}}+CD_{\mathrm{FS}}\right)$$

(15) $$W_{\mathrm{gross}}=P_{\mathrm{DS},\mathrm{out}}Q_{w}E_{G}$$

(16) $$PE_{\mathrm{DS}}=\frac{\left(P_{\mathrm{DS},\mathrm{in}}-P_{\mathrm{DS},\mathrm{out}}E_{\mathrm{px}}\right)Q_{\mathrm{DS},\mathrm{in}}}{E_{p,\mathrm{DS}}}$$

(17) $$PE_{\mathrm{FS}}=\frac{P_{\mathrm{FS},\mathrm{in}}Q_{\mathrm{FS},\mathrm{in}}}{E_{p,\mathrm{FS}}}$$

where $PE_{\mathrm{DS}}$ and $PE_{\mathrm{FS}}$ are the pumping energy at the DS and FS sides; *E*_p,DS_*_,_ E*_p,FS_*_,_ E*_G_, and *E*_px_ are the energy efficiencies of the DS pump, FS pump, electric generator, and pressure exchanger (PX), respectively. In this study, the values of *E*_G_ = 0.9, *E*_p,DS_ =0.88, *E*_p,FS_ = 0.88, and *E*_px_ = 0.92 were assumed based on the values in the literature [38,46].

## 3. Results and Discussion

### 3.1. RO Tests for the Two Modules

**Figure 3:**
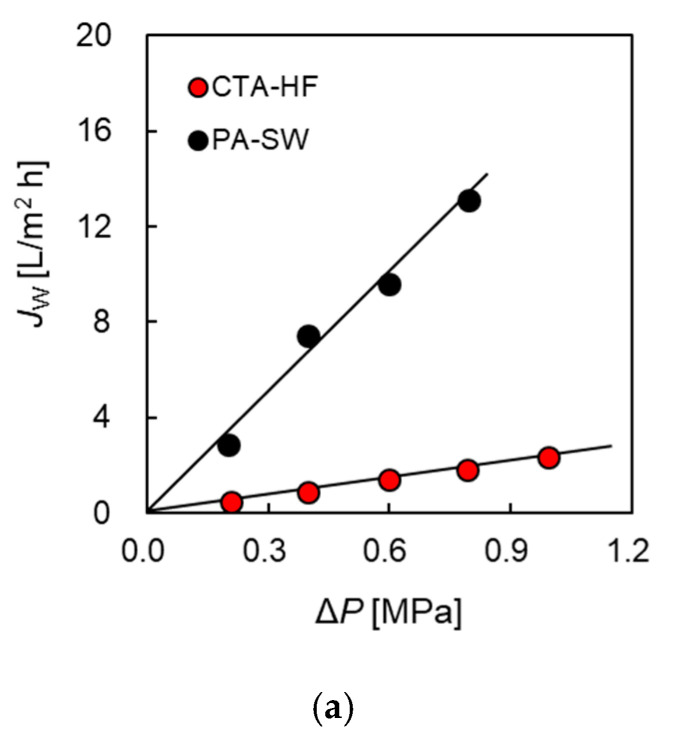

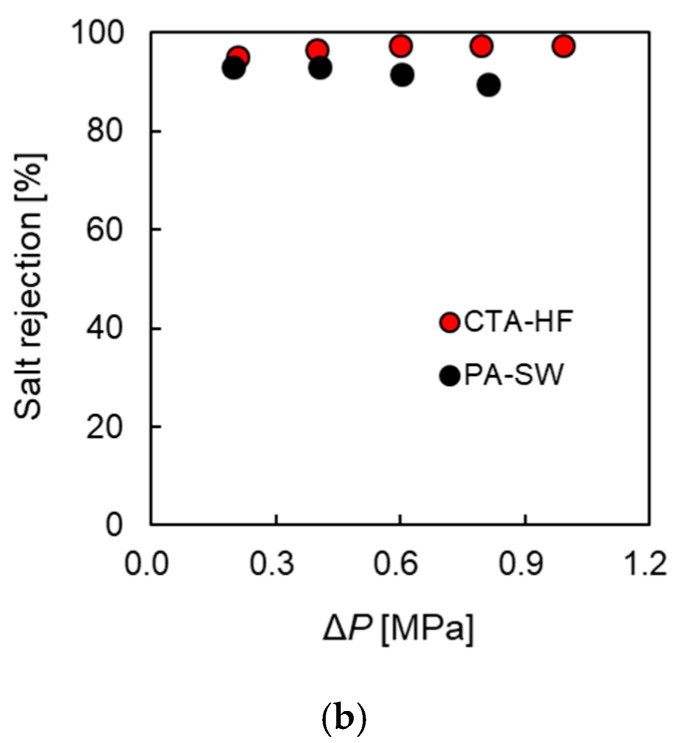
(**a**) Water flux and (**b**) salt rejection as a function of applied pressure in RO mode. Q_FS_: 4.0 L/min. Tap water and 500 ppm NaCl were used as feed solution in the measurement of water flux and salt rejection, respectively.

Figure 3a shows the water flux of PA-SW and CTA-HF plotted over the applied hydrostatic pressure (Δ*P*). The water flux increased with an increase in Δ*P*. The water permeability (*A* value) was obtained from the slope of the solid line calculated by fitting the data to Equation (1) and gives 0.65 × 10^−12^ and 4.6 × 10^−12^ [m s^−1^ Pa^−1^] for CTA-HF and PA-SW, respectively. The water permeability of PA-SW was approximately seven times higher than that of the hollow-fiber membrane module (CTA-HF). In general, the water permeability of a PA membrane is higher than that of a CTA membrane because the PA membrane has a thinner active layer than that of the CTA [4,42].

The salt rejection as a function of Δ*P*, using 500 ppm NaCl aqueous as the FS, is shown in Figure 3b. It was found that the salt rejection of CTA-HF was higher than that of PA-SW. The salt permeability (*B*) of CTA-HF and PA-SW was calculated by using Equation (2) from the salt rejection. The value of PA-SW was 2.23 × 10^−7^ [m s^−1^], approximately 13 times higher than that of CTA-HF (0.17 × 10^−7^ [m s^−1^]). This indicates that the CTA-HF membrane will have a lower DS leakage than the PA-SW membrane.

### 3.2. PRO Tests for the Two Modules

#### 3.2.1. Effect of FS Inlet Flow Rates on PRO Modules Performance

Generally, the performance of a PRO membrane module is lower than the theoretical value calculated in terms of Equation (9), owing to two factors: (a) external concentration polarization (ECP), which governs the water and solute permeation at the interface between the salt solution and the membrane surface, and (b) a decrease in between the DS and FS sides in the modules, owing to the water and salt permeation inside the module [10,12,13,25,36,47]. These phenomena decrease the PRO performance of the module and depend on the flow rates of the DS and FS. Hence, to obtain the optimum operating conditions for the two membrane modules, a systematic investigation of the effect of the inlet flow rate of DS and FS on PRO performance was conducted by measuring the water flux and the outlet concentration of DS and FS during the PRO tests, using 0.6 and 1.2 M NaCl as the DS, and applying a predetermined hydrostatic pressure difference.

##### Permeate Water Flux and Permeation Rate Versus FS Flow Rate

**Figure 4:**
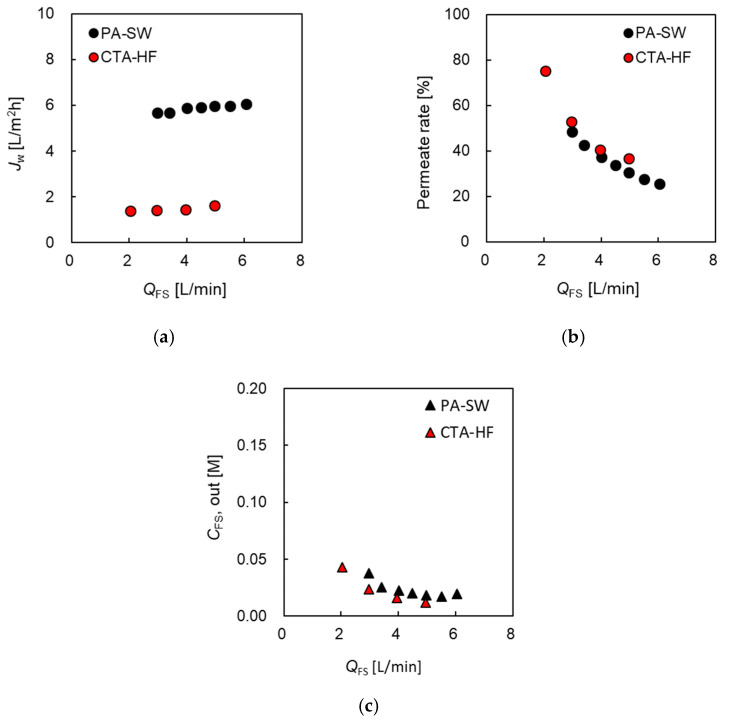
(**a**) Water flux, *J_w_*, and (**b**) FS permeation rate, *η*, and (**c**) the concentration of the module outlet at FS side (*C*_FS,out_), respectively, as a function of FS flow rate, *Q*_FS_. DS: 0.6 M NaCl. FS: treated tap water. Flow rate of DS (*Q*_DS_): 10.0 L/min. Hydrostatic pressure (Δ*P*): 1.2 MPa.

To evaluate the effect of concentrative internal concentration polarization (ICP) inside the support layer, and the increase in salt concentration in FS due to DS leakage (FS up-concentration) on the PRO performance of the two modules, the evaluation of the effect of *Q*_FS_ on *J_w_*, the permeation rate (*η*), and the concentration of the module outlet at the FS side were investigated and are shown in Figure 4. Here, 0.6 M NaCl and tap water were used as DS and FS, respectively. The flow rate of DS (*Q*_DS_) was set as 10.0 L/min in all the evaluations, which was the maximum DS flow rate of the evaluation system. Δ*P* was set as 1.2 MPa, which is almost half of the theoretical osmotic pressure difference between the DS and FS. Figure 4a shows that *J_w_* of PA-SW gradually increased from 5.6 at 3.0 L/min to 6.1 L/m^2^h at 6.0 L/min with increasing *Q*_FS_, while that of CTA-HF from 1.4 at 2.0 L/min to 1.6 L/m^2^h at 5.0 L/min. These results indicate that *J_w_* of the former module was more than 4 times higher than that of the latter, though the water permeability of the former was more than 13 times higher than that of the latter. The slight increase in *J_w_* of both modules with increasing *Q*_FS_ indicates that the effect of ICP on the PRO performance is almost negligible at high *Q*_FS_. Figure 4b indicates that the permeate rate of the two modules decreased with increasing *Q*_FS_ and the value of CTA-HF decreased from 75% at 2.0 L/min to less than 40% at 5 L/min, and that of PA-SW from 48% at 3.0 L/min to 26% at 6 L/min. Figure 4c shows that FS outlet concentration (*Q*_FS,out_) of both the modules was less than 0.05 M and decreased with increasing *Q*_FS_. Increasing *Q*_FS_ helped to flush the salt in the FS that permeated from the DS side to the FS membrane interface. For a CTA-HF module, a permeation rate of less than 70% was required to mitigate the FS up-concentration [35]. $C_{\mathrm{FS},\mathrm{out}}$ of the CTA-HF, was slightly lower than that of PA-SW, although the permeate rate of the former was slightly higher than that of the latter. This is because the *B* value of the former is significantly lower than that of the latter. From the results shown in Figure 4b,c, an FS flow rate of more than 5 L/min is sufficient for the two modules to mitigate the effect of FS up-concentration on the PRO performance.

**Figure 5:**
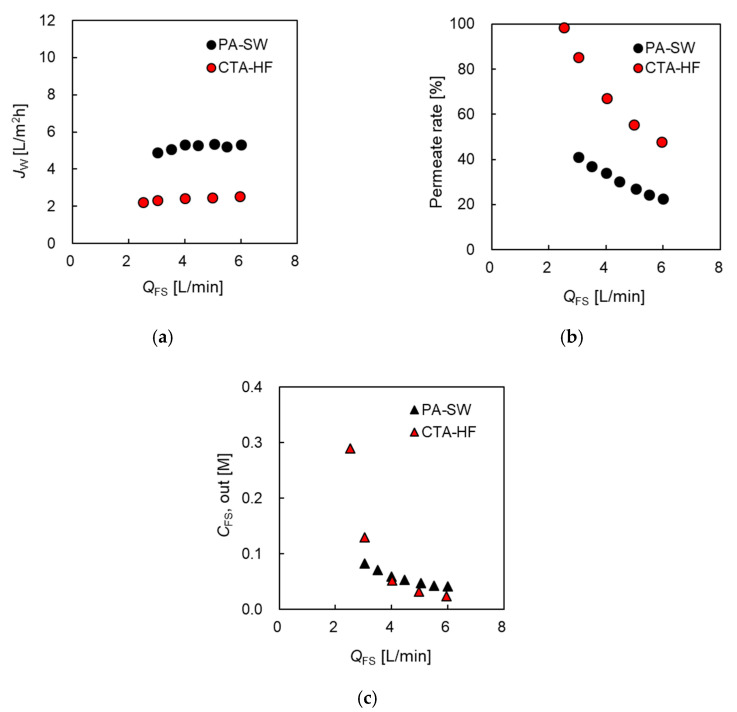
(**a**) Water flux, *J_w_*, and (**b**) FS permeation rate, *η*, and (**c**) the concentration of the module outlet at FS side (*C*_FS,out_), respectively, as a function of FS flow rate, *Q*_FS_. DS: 1.2 M NaCl. FS: treated tap water. *Q*_DS_: 10.0 L/min. Δ*P*: 1.6 MPa for PA-SW module and 2.5 MPa for CTA-HF module.

Figure 5 shows *J_w_* and *η*, and *Q*_FS,out_ as a function of *Q*_FS_ in the case of 1.2 M NaCl as DS. Here, Δ*P* for CTA-HF was 2.5 MPa, which is almost equal to Δ*π*. There is no information about the pressure resistance for PA-SW. Hence, the PRO test using PA-SW was performed at a lower pressure than that using CTA-HF, i.e., 1.6 MPa.

In the case of 1.2 M DS, *J_w_* of CTA-HF at 6.0 L/min of *Q*_FS_ was 1.56 times higher compared to that in the case of 0.6 M though Δ*P* increased from 1.2 to 2.5 Mpa, because the increase in Δ*π* as the driving force of the water flux, as shown in Figure 5a. On the contrary, *J_w_* of PA-SW showed a low value of 85% of *J_w_* in the case of 0.6 M NaCl. This is because the increase in the effective osmotic pressure difference in PA-SW did not increase linearly, as mentioned in Section 3.2.2. As shown in Figure 5b, the permeation rate in CTA-HF reached 100% at the lowest *Q*_FS_ (<2.5 L/min), and *C*_FS,out_ of the module had a high value of approximately 0.3 M, as shown in Figure 5c. Although the *J_w_* of CTA-HF is lower than that of PA-SW, the total membrane area of the former is more than four times larger than that of the latter; hence, the permeate water flow from the FS to the DS sides in the former module was larger than that in the latter. Therefore, an FS flow rate of 2.5 L/min is not sufficient for CTA-HF to prevent FS up-concentration. *C*_FS,out_ decreased dramatically as *Q*_FS_ increased, and permeation rate decreased to less than 50% at 6.0 L/min of *Q*_FS_, while that of PA-SW decreased from 40% to 20% at 6.0 L/min of *Q*_FS_. Hence, these data indicate that 5.0 a *Q*_FS_ of is sufficient for both modules to mitigate FS up-concentration even at a high DS concentration of 1.2 M.

##### Permeate Water Flux and Dilution Factor Versus DS Flow Rate

**Figure 6:**
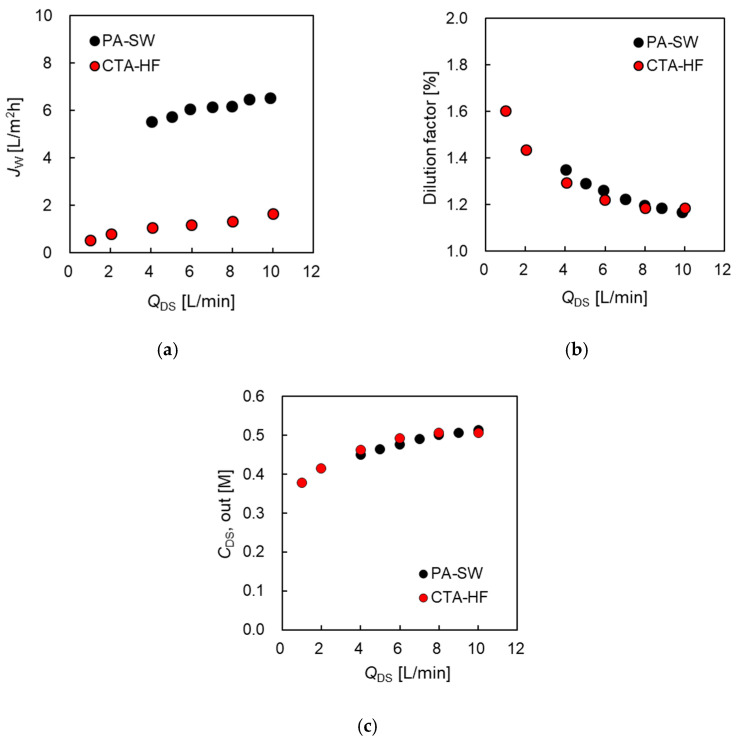
(**a**) Water flux, *J_w_*, and (**b**) dilution factor, *φ*, and (**c**) the concentration of the module outlet at DS side (*C*_DS,out_), respectively, as a function of DS flow rate, *Q*_DS_. DS: 0.6 M NaCl. FS: treated tap water. *Q*_FS_: 5.0 L/min. Δ*P*: 1.2 MPa.

The effect of the DS flow rate (*Q*_DS_) on the PRO performance was investigated by measuring the PRO performance of the two modules at various *Q*_DS_ to evaluate the effect of dilutive external concentration polarization (dECP) at the DS side and the decrease in the salt concentration in the module inside the DS side. Figure 6 shows *J_w_*, dilution factor (*φ*), and the concentration of the module outlet at the DS side (*Q*_DS,out_) as a function of *Q*_DS_, where DS and FS are 0.6 M NaCl and tap water, respectively. *Q*_FS_ was set as 5.0 L/min, and Δ*P* was 1.2 MPa, which is nearly equal to the osmotic pressure difference between DS and FS.

Figure 6a shows that *J_w_* increased from 0.53 at 1.0 L/min to 1.7 L/m^2^h at 10 L/min of *Q*_DS_ for CTA-HF and from 5.3 at 4.0 L/min to 6.5 L/m^2^h at 10 L/min for PA-SW as *Q*_DS_ increased, even as it approached the maximum flow rate of the pump. As *φ* approaches 1, it indicates that *Q*_DS,out_ is equal to the DS flow rate at the inlet. In this case, *φ* decreased from 1.58 at 1.0 L/min to 1.18 at 10 L/min (CTA-HF) and from 1.33 at 4 L/min to 1.17 at 10 L/min (PA-SW), as shown in Figure 6b. In the case of CTA-HF, *C*_DS,out_ increased from 0.38 M at 1.0 L/min to 0.51 M at 10 L/min, 63% and 85% of the DS inlet concentration, respectively. In the case of PA-SW, *C*_DS,out_ increased from 0.45 M and 0.51 M, 75% and 85% of the DS inlet concentration, respectively, as *Q*_DS_ increased, as shown in Figure 6c. This indicates that a 10 L/min of DS flow rate is not sufficient for both modules to be negligible in the DS dilution effect by the permeate water flow from the FS side.

**Figure 7:**
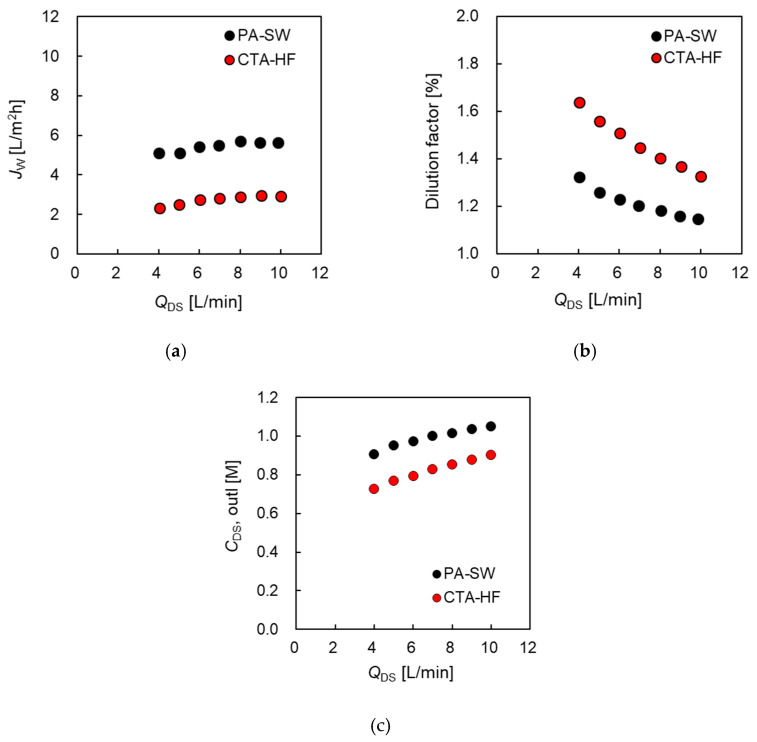
(**a**) Water flux, *J_w_*, and (**b**) dilution factor, *φ*, and (**c**) the concentration of the module outlet at DS side (*C*_DS,out_), respectively, as a function of DS flow rate, *Q*_DS_. DS: 1.2 M NaCl. FS: treated tap water. Q_FS_: 5.0 L/min. Δ*P*: 1.6 MPa for PA-SW module and 2.5 MPa for CTA-HF module.

When using 1.2 M NaCl as DS, Figure 7a shows that *J_w_* increased from 2.32 at 4.0 L/min to 2.9 L/m^2^h at 10 L/min of *Q*_DS_ for CTA-HF. For PA-SW, *J_w_* increased from 5.1 at 4.0 L/min to 5.6 L/m^2^h at 10 L/min of *Q*_DS._ Similar to the case of FS flow rate change, *J_w_* of CTA-HF was higher compared to that in the case of 0.6 M, even though Δ*P* increased; however, that of PA-SW had a lower value. *φ* decreased from 1.65 at 4.0 L/min to 1.33 at 10 L/min (CTA-HF) and from 1.32 at 4 L/min to 1.14 at 10 L/min (PA-SW), as shown in Figure 7b. *C*_DS,out_ increased from 0.73 M at 4.0 L/min to 0.90 M at 10 L/min for CTA-HF, 61% and 75% of the DS inlet concentrations, respectively, and from 0.90 M at 4.0 L/min to 1.0 M at 10 L/min for PA-SW, 75% and 83% of the DS inlet concentration, respectively, as shown in Figure 7c. Even at the highest DS flow rate of the evaluation system (10 L/min), especially in the case of CTA-HF, *φ* showed a high value (more than 1.3), and *C*_DS,out_ was much lower than the DS inlet concentration. This is because the permeate water flow of the module for CTA-HF was approximately two times higher than that of PA-SW; although, the water flux of the former was approximately half that of the latter at 10 L/min, indicating that the dilution effect to reduce the PRO performance cannot be negligible, especially for CTA-HF under the test conditions.

#### 3.2.2. PRO Performance of the Two Modules as a Function of Applied Pressure

**Figure 8:**
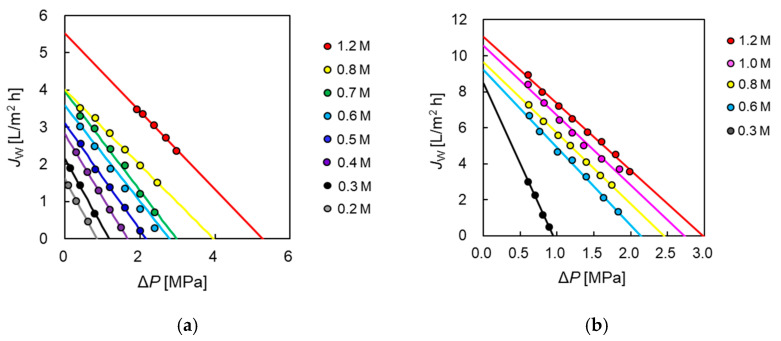
Water flux, *J_w_*, as a function of applied hydraulic pressure difference, Δ*P*, at *Q*_DS_ = 10.0 L/min and *Q*_FS_ = 5.0 L/min: (**a**) PA-SW and (**b**) CTA-HF membrane module.

Figure 8a,b shows the relationship between *J_w_* and Δ*P* of CTA-HF and PA-SW, respectively, at various inlet DS concentrations (*C*_DS_), where the flow rates of DS and FS were 10.0 and 5.0 L/min, respectively. As shown in Equation (1) and Figure 1, the measured water flux of both modules decreased with increasing Δ*P* because Δ*P* facilitates water movement from the DS to FS sides. The measured water flux increased with increasing *C*_DS_, owing to the increase in the driving force of water permeation and the osmotic pressure difference.

Although relationship between *J_w_* and Δ*P*, given by Equation (1) indicates a linear relationship, in general, the actual *J_w_* in a PRO system decreases along the parabolic curve with increasing Δ*P* [29]. However, the obtained water flux of both modules decreased almost linearly with increasing Δ*P*. This is primarily because the PRO tests in this study were performed at high DS and FS flow rates to minimize the effect of the DS dilution, dECP at the DS side by the permeate water flux, and the concentrated ICP inside the module by DS leakage, although these effects were not negligible, owing to the limitation of the pump performance, as shown in Figure 7. From the experimental results, the experimental water flux, $J_{w,\exp}$, of the two modules under PRO conditions can be expressed in terms of a simple linear approximation as an empirical line [35]:

(18) $$J_{w,\;\exp}=\;A^{\mathrm{PRO}}\left(\Delta \pi_{\mathrm{eff}}-\Delta P\right)$$

**Figure 9:**
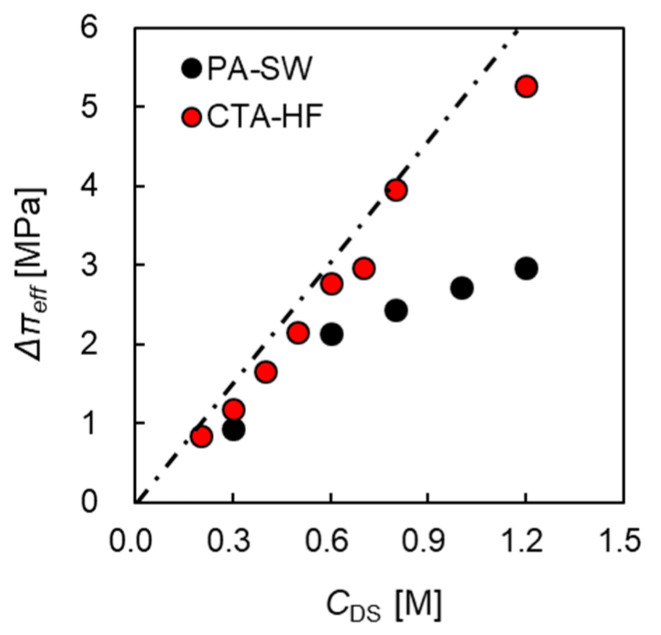
Effective osmotic pressure difference, $\Delta \pi_{\mathrm{eff}}$, as a function of DS concentration, *C*_DS._ FS: treated tap water. *Q*_DS_: 10.0 L/min. *Q*_FS_: 5.0 L/min.

Here, the pseudo-water permeability in PRO mode ($A^{\mathrm{PRO}}$) and effective osmotic pressure difference ($\Delta \pi_{\mathrm{eff}}$) were obtained from the slope and intersection of the *x*-axis of the empirical line shown in Figure 8. The relationship between $\Delta \pi_{\mathrm{eff}}$ and *C*_DS_ is shown in Figure 9. The broken line represents the theoretical osmotic pressure difference (Δπ) calculated by using the van’t Hoff equation. This figure shows that $\Delta \pi_{\mathrm{eff}}$ increased as *C*_DS_ increased, and $\Delta \pi_{\mathrm{eff}}$ of the two modules was lower than Δπ. Additionally, PA-SW exhibited a greater deviation between $\Delta \pi_{\mathrm{eff}}$ and Δπ, especially at high *C*_DS_. In contrast, CTA-HF shows a small deviation between the two values, and from the relationship between $\Delta \pi_{\mathrm{eff}}$ and *C*_DS_ shown in Figure 9, $\Delta \pi_{\mathrm{eff}}$ inside the module in the PRO test is estimated by using Equation (19), and *α*_mod_ is calculated as 0.88:

(19) $$\Delta \pi_{\mathrm{eff}}=\alpha_{\mathrm{mod}}\Delta \pi$$

The small deviation between Δπ and $\Delta \pi_{\mathrm{eff}}$ for CTA-HF despite *Q*_DS,out_ being 85% of the inlet concentration in the case of 0.6 M NaCl shown in Figure 6c indicates that the primary cause of the deviation will be the DS dilution, and the effect of the dilutive ECP on PRO performance will be negligible for the HF type module.

The possible reasons that the dilutive ECP (dECP) will be negligible are as follows: A hollow-fiber-type membrane element has an approximately 10-times-larger surface area (than SW type) in the case of an RO module [42]. This implies that the water flux per membrane area of an HF module is much lower than that of an SW module, even if the two modules have the same water flux per module. Hence, the effect of dECP on the DS side will be lower because of the small water flux per membrane area of the HF module. Additionally, the cross-winding arrangement of more than several thousands of hollow fibers in an HF module allows a uniform flow, which minimizes the concentration polarization in the shell side (the DS side in this study) of the module [42].

The other possible reasons for the deviations Δπ and $\Delta \pi_{\mathrm{eff}}$ are that the smaller *B* value of CTA-HF compared to PA-SW will give a lower concentrative ICP inside the support layer of the membrane.

**Figure 10:**
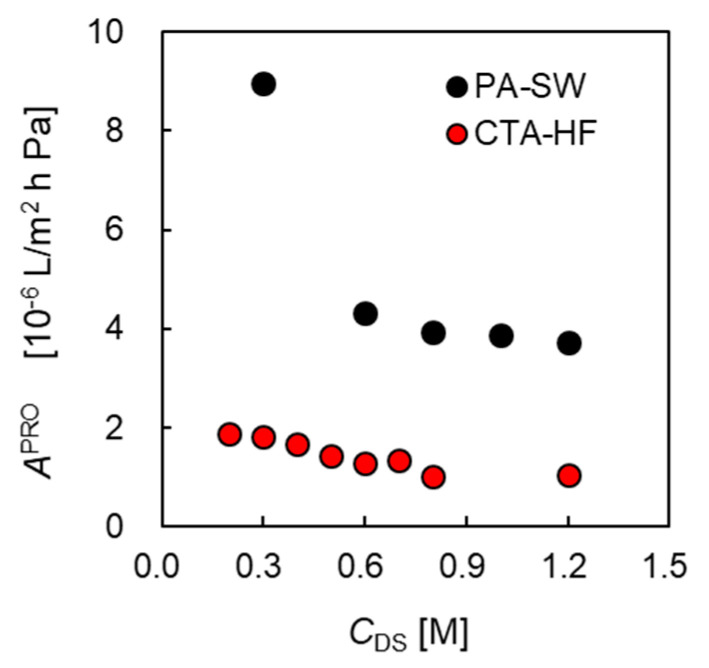
Apparent water permeability coefficient, $A^{\mathrm{PRO}}$, as a function of DS concentration, *C*_DS_. FS: treated tap water. *Q*_DS_: 10.0 L/min. *Q*_FS_: 5.0 L/min.

Figure 10 shows the apparent water permeation coefficient $A^{\mathrm{PRO}}$ as a function of *C*_DS_. The $A^{\mathrm{PRO}}$ of CTA-HF decreased gradually from 1.9$\times 10^{-6}$ at 0.2 M NaCl to 1.0 $\times 10^{-6}$ [L/m^2^ h Pa] at 1.2 M of *C*_DS_, which is similar to the decrease of the CTA-HF reported in a previous study [43] named as M-HF in Table 2. $A^{\mathrm{PRO}}$ of PA-SW decreased drastically from 9.0 at 0.3 M of *C*_DS_ to 3.7 $\times 10^{-6}$ [L/m^2^ h Pa] at 1.2 M DS. As shown in Figure 9 and Figure 10, the drastic decrease in $\Delta \pi_{\mathrm{eff}}$ and $A^{PRO}$ of PA-SW is due to the concentrated ICP inside the support layer of the PA membrane involving the DS leakage because the membrane has lower salt rejection than the CTA membrane, as shown in Figure 3b. Another factor may be the complex FS flow channel structure of the PRO SW module in a U-fashion [48]. It may be difficult for the channel structure to flush the DS leakage at a high *C*_DS_.

#### 3.2.3. Comparison of PRO Module Performance between CTA-HF and PA-SW

The volumetric power density of the hollow-fiber-type module (CTA-HF) and spiral-wound type of membrane module (PA-SW) were compared to investigate the feasible operating conditions for generating energy by using the PRO technique. In this section, a detailed comparison was performed to evaluate the volumetric power density as a function of the applied hydrostatic pressure difference, and the maximum gross power, maximum net power output, and pumping energy for feeding DS and FS as a function of the flow rates of the DS and FS.

##### Volumetric Gross Power Density and Applied Pressure Difference

**Figure 11:**
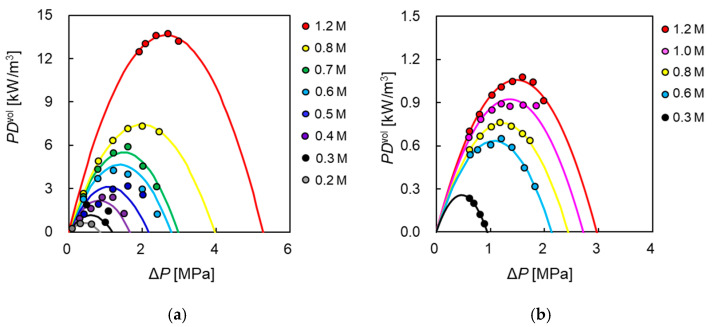
Volumetric gross power density, *PD*^vol^*,* as a function of applied hydraulic pressure difference, Δ*P*, membrane module: (**a**) PA-SW and (**b**) CTA-HF with FS: treated tap water. *Q*_DS__:_ 10.0 L/min_._ *Q*_FS_: 5.0 L/min.

Figure 11a,b shows the volumetric gross power density (*PD*^vol^) of PA-SW and CTA-HF in relation to Δ*P*, respectively. Here, each circle represents the experiments obtained under the operating conditions where *Q*_DS_ and *Q*_FS_ are 10 and 5 L/min, respectively, and each curve is the calculation obtained by substituting $A^{\mathrm{PRO}}$ and $\Delta \pi_{\mathrm{eff}}$ into Equations (9) and (18). These figures indicate that the *PD*^vol^ of both modules increased with increasing Δ*P*, and they reached a maximum value at approximately half of $\Delta \pi_{\mathrm{eff}}$ at each *C*_DS_. The maximum volumetric gross power density ($PD_{\max}^{\mathrm{gross}}$) and the applied hydrostatic pressure difference at the point that indicates $PD_{\max}^{\mathrm{gross}}$ increased when *C*_DS_ increased, as predicted by Equation (18). Compared to $PD_{\max}^{\mathrm{gross}}$ of PA-SW and CTA-HF, in the case of 1.2 M NaCl (SWRO brine level) as DS, $PD_{\max}^{\mathrm{gross}}$ of CTA-HF was 13.6 kW/m^3^ at 2.6 MPa of Δ*P*, and that of PA-SW was 1.1 kW/m^3^ at 1.5 MPa of Δ*P*. Hence, CTA-HF showed 12 times higher $PD_{\max}^{\mathrm{gross}}$ than PA-SW. In the case of 0.6 M NaCl (seawater level) as DS, $PD_{\max}^{\mathrm{gross}}$ of CTA-HF was 4.7 kW/m^3^ (Δ*P* = 1.4 MPa), and that of PA-SW is 0.63 kW/m^3^ (Δ*P* = 1.1 MPa), indicating 7.5 times higher $PD_{\max}^{\mathrm{gross}}$ of CTA-HF. This is because of the difference in the packing density (the ratio of membrane area to module volume) between the two module configurations. The packing densities of CTA-HF and PA-SW were 6.75 $\times \;10^{3}$ and 464 1/m, respectively; thus, that of CTA-HF was 14 times higher than that of PA-SW. Therefore, the permeate water flux of CTA-HF is higher than that of PA-SW, even though the water flux per unit membrane area of the latter is approximately three times higher than that of the former, as shown in Figure 6a, and CTA-HF has only 30% of the module volume compared to PA-SW. The total membrane area of PA-SW (15.3 m^2^) is approximately 40% that of a conventional RO SW module (37 m^2^). This is owing to the thicker tricot fabric spacer at the FS side and larger dead membrane area due to the presence of additional glue lines along the central line of the membranes to fabricate in a U-shaped fashion [48], compared to a conventional RO SW module.

**Figure 12:**
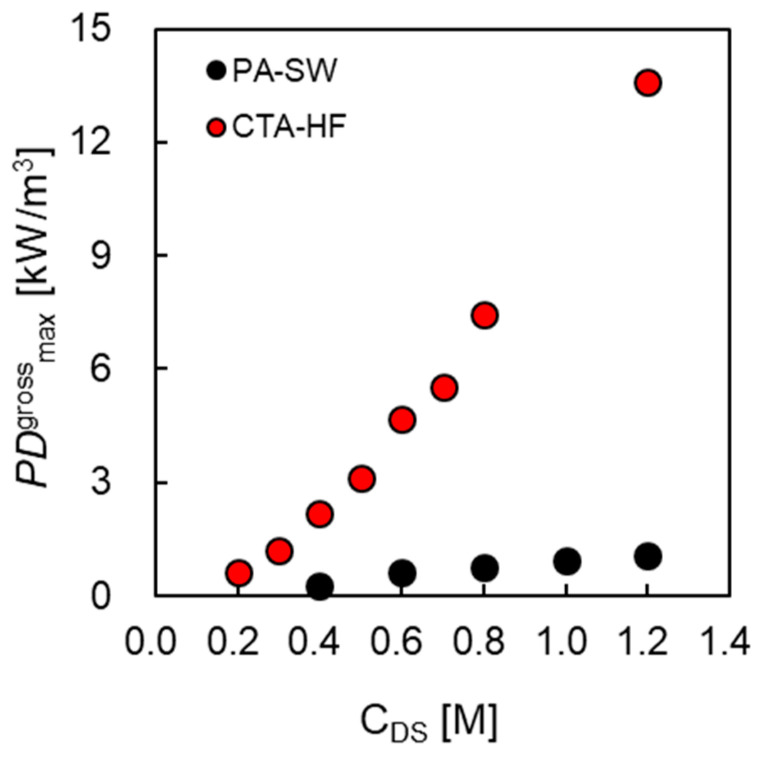
The maximum gross power output ($PD_{\max}^{\mathrm{gross}}$) of CTA-HF and PA-SW as a function of *C*_DS_. FS: treated tap water. *Q*_DS:_ 10.0 L/min. *Q*_FS_: 5.0 L/min.

To investigate the relationship between $PD_{\max}^{\mathrm{gross}}$ and DS concentration in greater detail, Figure 12 shows the $PD_{\max}^{\mathrm{gross}}$ of CTA-HF and PA-SW as a function of *Q*_DS_. The $PD_{\max}^{\mathrm{gross}}$ of CTA-HF increased in an approximate quadratic curve with increasing *C*_DS_. In the theoretical Equations (1) and (9), the power output of PRO becomes a quadratic curve of *C*_DS_ because the power output is the product of the water flux and the osmotic pressure difference, and these two factors depend linearly on *C*_DS._ Conversely, the degree of the increase in $PD_{\max}^{\mathrm{gross}}$ of PA-SW was smaller than that of CTA-HF, indicating that there is a large difference in $PD_{\max}^{\mathrm{gross}}$ between the two modules, especially at high DS concentrations. The primary causes of this difference are the difference in $\Delta \pi_{\mathrm{eff}}$ and $A^{PRO}$ at high DS concentrations and the packing density between the two modules.

##### Relationship between Net Power Output and Feed Flow Rates of DS and FS

**Figure 13:**
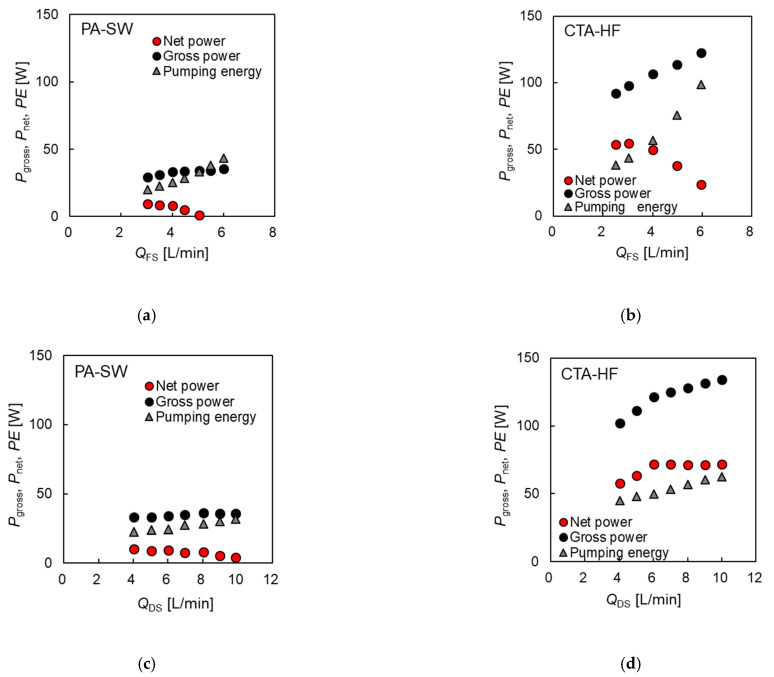
The gross power (*P*_gross_), net power (*P*_net_), and pumping energy (*PE*) per a module as a function of flow rates of DS and FS, using PA-SW (**a**) and (**c**) and CTA-HF (**b**) and (**d**).

The theoretical power output of the membrane module was calculated from the water flux and applied pressure difference. However, in an actual PRO plant, the efficiency of the pumps and PX used for PRO operation must be carefully considered. Hence, in this section, the gross power, net power per module, and pumping energy in the PA-SW and CTA-HF systems were compared, using 1.2 M NaCl as DS, at various flow rates of DS and FS. Here, tap water was used as FS, and Δ*P* for PA-SW and CTA-HF were 1.6 and 2.5 MPa, respectively. Figure 13a,b shows the gross power (*P*_gross_), net power (*P*_net_), and pumping energy (*PE*) per module for PA-SW and CTA-HF as a function of FS flow rate. Figure 13a shows that *P*_gross_ of PA-SW increased from 28 to 35 W at 6 L/min, indicating a 1.25-fold increase from 3 to 6 L/min *Q*_FS_, while *CE* increased rapidly from 20 to 44 W (2.2-fold increase). The *P*_net_ of the module decreased from 9.3 W and showed negative values at flow rates of more than 5 L/min. Hence, *P*_net_ had a maximum value of 9.3 W at 3.0 L/min of *Q*_FS_ under the test conditions. Meanwhile, as shown in Figure 13b, *P*_gross_ of CTA-HF increased from 93 to 123 W, a 1.3-fold increase with increasing *Q*_FS_ from 3 to 6 L/min. The *CE* increased rapidly from 43 to 98 W (2.3-fold increase). *P*_net_ increased from 54 W and reached a maximum value of 55 W at 3 L/min of *Q*_FS_, and then decreased to 23 W at 6 L/min of *Q*_FS_. The maximum net power of the CTA-HF is more than five times that of the PA-SW. The changes in *P*_gross_, *P*_net_, and *CE* with DS flow rate are shown in Figure 13c,d. The *P*_gross_ of PA-SW increased from 33 W at 4 L/min to 36 W at 10 L/min, indicating a 1.09-fold increase with increasing *Q*_DS_, while *CE* increased rapidly from 22 to 32 W (1.45-fold increase). The *P*_net_ of the module decreased from the maximum value of 11 W at 4.0 L/min and showed negative values at flow rates of more than 9 L/min. For CTA-HF, *P*_gross_ increased from 102 W at 4 L/min to 128 W at 10 L/min, indicating a 1.25-fold increase with increasing *Q*_DS_, while *CE* increased rapidly from 43 to 63 W (1.46-fold increase). Thus, *P*_net_ of the module increased from 58 W at 4 L/min and reached a maximum of 72 W at 6 L/min and almost a constant value of 71 W. From these results, the dependence of *Q*_FS_ for both modules on *CE* is higher than that of *Q*_DS_. The rapid increase in *CE* with increasing *Q*_FS_ for PA-SW is caused by the complex flow channel in the dense tricot spacer with high pressure resistance at the FS side mentioned above. For CTA-HF, small-diameter hollow fibers are used in the module. The evaluation indicates that as the flow rates of DS and FS increased for both modules, *P*_gross_ increased. However, the rapid increase in *CE* for PRO operation with increasing flow rates of DS and FS reduced *P*_net_. PA-SW demonstrates a maximum value of *P*_net_ at lower DS and FS flow rates than the operating conditions of the PRO test in this study. For CTA-HF, the optimal value of *Q*_DS_ and *Q*_FS_ are 6.0 and 3.0 L/min, respectively, under the test conditions. The rapid increase in *CE* with increasing *Q*_FS_ is the primary cause of the reduction in the net power of the PRO system, especially for CTA-HF. Hence, a flow channel structure with a low pressure drop is necessary to increase the PRO power output.

#### 3.2.4. Comparison of PRO Performance between Spiral-Wound and Hollow-Fiber Modules

To compare the PRO module performances with different configurations, such as spiral wound (SW) and hollow fiber (HF), a comparison using volumetric-based power outputs, as well as membrane area-based power density, is needed to design a full-scale PRO plant. Table 2 shows the maximum power density per unit membrane area ($PD_{\max}^{\mathrm{area}}$), maximum power density per unit volume ($PD_{\max}^{\mathrm{vol}}$), test conditions, and module dimensions of the modules tested in this study and those in the literature. Kim et al. [37] evaluated the PRO performance of a prototype PRO SW module (M-SW) with an effective membrane area of 29 m^2^ of the TFC membrane. The dimensions of the module were 0.2 m (8 in.) in diameter and 1 m in length; hence, the module volume was estimated to be 31.4 × 10^−3^ m^3^. $PD_{\max}^{\mathrm{area}}$ was reported as 1.0 W/m^2^; thus, $PD_{\max}^{\mathrm{vol}}$ of the module was estimated as 0.92 kW/m^3^, using 0.6 M NaCl as DS at 0.98 MPa of Δ*P*. In comparison with M-SW, PA-SW in this study demonstrated 40% higher $PD_{\max}^{\mathrm{area}}$ (1.40 W/m^2^) and 74% of $PD_{\max}^{\mathrm{vol}}$ (0.68 kW/m^3^). The smaller $PD_{\max}^{\mathrm{vol}}$ of PA-SW was due to approximately half of the packing density, owing to the smaller effective membrane area of PA-SW compared to M-SW.

Higa and Yasukawa et al. [35,43] evaluated the PRO performance of a CTA-HF module (M-HF). The effective membrane area and volume of the module were 72 m^2^ and 9.27 × 10^−3^ m^3^, respectively. The packing density of M-HF was calculated as 7769 1/m, the highest value in all the modules listed in Table 2. $PD_{\max}^{\mathrm{area}}$ of the module increased from 0.14 at 0.5 M to 0.44 W/m^2^ at 0.9 M NaCl as DS, and $PD_{\max}^{\mathrm{vol}}$ changed from 1.09 at 0.5 M to 3.39 W/m^2^ at 0.9 M NaCl. The effective membrane area of M-HF was 72 m^2^, which is higher than that of CTA-HF. This is because the outer diameter and number of HF using M-HF were 160 μm and 214,000, respectively, while those of CTA-HF were 170 μm and 187000. Hence, the packing density of the former is higher than that of CTA-HF. In a comparison of the power output between the two HF modules, CTA-HF shows approximately three times higher $PD_{\max}^{\mathrm{area}}$ and $PD_{\max}^{\mathrm{vol}}$ than M-HF. This is owing to higher A value (0.65 × 10^−12^ [m s^−1^ Pa^−1^]) of the former than that of the latter (0.25 × 10^−12^ [m s^−1^ Pa^−1^]) primarily because of the thinner membrane of CTA-HF (40 μm) compared to that of M-HF (50 μm) [35].

In a comparison of the power output between modules with different configurations, $PD_{\max}^{\mathrm{area}}$ of PA-SW was 2.03-fold higher at 0.6 M NaCl and 1.16-fold higher at 1.2 M NaCl than that of CTA-HF due to the higher water flux of PA-SW. Conversely, in a comparison using the volumetric-based power output, CTA-SW showed 6.87-fold higher $PD_{\max}^{\mathrm{vol}}$ at 0.6 M NaCl than PA-SW did. The difference in the $PD_{\max}^{\mathrm{vol}}$ between the two modules increased with increasing DS concentration, and CTA-HF had 12.0-fold higher $PD_{\max}^{\mathrm{vol}}$ at 1.2 M NaCl as DS. The higher volumetric-based power density of CTA-HF is due to its higher packing density than that of PA-SW. Moreover, at high DS concentrations, the lower *B* value of the CTA-HF reduces concentrative ICP due to DS leakage, and the lower water permeate per unit membrane area of HF membranes reduces the effect of dECP at the DS side on the PRO performance. To achieve a high volumetric-based power output for a spiral-wound membrane module, an increase in the packing density to optimize the FS flow channel structure is required. In addition, a low *B* value to reduce DS leakage and high DS flow rates to decrease dECP are needed to improve the PRO performance of an SW module at high DS concentrations.

In this study, we compared the PRO performance between CTA-HF and PA-SW, using NaCl solutions of various concentrations as model DS. In a real PRO application, DS and FS will contain foulant substances, and there will be a difference in the effect of the foulants on the PRO performance between the two module configurations. For example, the more complex channel structure of the U fashion at the FS side of PA-SW will have a greater impact on the long-term stability than CTA-HF. Additionally, DS and FS contain not only monovalent ions such as Na^+^ and Cl^-^ ions but also bivalent ions such as Mg^2+^, Ca^2+^, and SO_4_^2-^ ions. The difference in the reverse flux between the monovalent ions and the bivalent ions will affect the FS up-concentration on the PRO performance and also the difference in the membrane fouling by forming aggregates between the bivalent ions and foulants in FS, such as humic acid [49].

## 4. Conclusions

In this study, permeate water flux in PRO mode, using two pilot-scale PRO membrane modules with different configurations, a five-inch cellulose triacetate hollow-fiber membrane module (CTA-HF) and eight-inch polyamide spiral-wound membrane modules (PA-SW), was measured by changing the DS concentration, the applied hydrostatic pressure difference, and the flow rates of the DS and FS, to obtain the optimum operating conditions in the PRO configuration. The following conclusions were drawn from this study:

The water permeability of PA-SW was approximately seven times higher than that of CTA-HF. In comparison, the salt permeability of PA-SW was approximately 13 times higher than that of CTA-HF, indicating that the CTA-HF membrane will have a lower DS leakage than PA-SW.

An FS flow rate of more than 5 L/min is sufficient for the two modules to mitigate the effect of FS up-concentration on PRO performance. However, 10 L/min of DS flow rate cannot make the dilution effect negligible, especially at high DS concentrations (*C*_DS_).

PA-SW showed a significant deviation between the theoretical and effective osmotic pressure differences obtained from the water flux vs. the applied hydrostatic pressure curves in the PRO test, especially at high *CDSs*. In contrast, the CTA-HF showed a small deviation between the two values.

The FS flow rate’s (*Q*_FS_) dependence for both modules on the pumping energy for PRO operation is higher than that for DS flow rate. The rapid increase in the pumping energy with increasing *Q*_FS_ is due to the complex U-shaped flow channel in the dense tricot spacer at the FS side for PA-SW and the small diameter of hollow fibers used in CTA-HF.

The maximum power density per unit membrane area ($PD_{\max}^{\mathrm{area}}$) of PA-SW was 2.03-fold higher at 0.6 M NaCl and 1.16-fold higher at 1.2 M NaCl than that of CTA-HF, owing to the higher water flux of PA-SW. Conversely, in a comparison using the maximum power density per unit volume ($PD_{\max}^{\mathrm{vol}}$), CTA-SW showed a 6.87-fold higher $PD_{\max}^{\mathrm{vol}}$ at 0.6 M. The difference in the $PD_{\max}^{\mathrm{vol}}$ between the two modules increased with the increasing DS concentration, and CTA-HF had 12.0-fold higher $PD_{\max}^{\mathrm{vol}}$ at 1.2 M NaCl as DS than PA-SW did. This is owing to the high packing density of the CTA-HF.

## Figures and Tables

**Table 1:** Specifications of the cellulose triacetate hollow-fiber (CTA-HF) and polyamide spiral-wound (PA-SW) membrane modules used in this study.

	CTA-HF	PA-SW
Active layer of membrane	Cellulose triacetate	polyamide
Number of ports	4	4
Module diameter (mm)	136	203.2
Module length (mm)	683	1016
Inner diameter of hollow fiber, d_in_ (μm)	90	-
Outer diameter of hollow fiber, d_out_ (μm)	170	-
Number of hollow fibers	187,000	-
Membrane area, (m^2^)	67	15.3
Module volume, (m^3^)	9.92 × 10^−3^	3.3 × 10^−2^
Packing density (m^2^/m^3^)	6754	464

**Table 2:** Comparison between the dimensions and power densities of PRO modules from the literature and the current study.

Module	Type	*S_m_* (m^2^)	V_m_ (m^3^) × 10^−3^	β (1/m)	DS(M NaCl)	Δ*P* at *PD*_max_ (M Pa)	$PD_{\max}^{\mathrm{area}}$ (W/m^2^)	$PD_{\max}^{\mathrm{vol}}$ (kW/m^3^)	Reference
M-SW	SW	29	31.4	923	0.6	0.98	1.0	0.92	[37]
M-HF	HF	72	9.27	7769	0.5	0.94	0.14	1.09	[35]
					0.6	1.1	0.17	1.32	[43]
					0.8	1.7	0.35	2.74	[43]
					0.9	1.8	0.44	3.39	[43]
PA-SW	SW	15.3	31.4	487	0.6	1.2	1.40	0.68	This study
					0.8	1.2	1.64	0.80	
					1.2	1.6	2.33	1.13	
CTA-HF	HF	67	9.92	6770	0.5	1.6	0.47	3.21	This study
					0.6	1.2	0.69	4.67	
					0.8	2.0	1.09	7.36	
					1.2	3.0	2.01	13.61	

M = module, FS = tap water, SW = spiral wound, HF = hollow fiber.

## References

[1] IEA (2014). CO2 Emission from Fuel Combustion—Highlights.

[2] Kruyt B., Van Vuuren D.P., De Vries H.G.M., Geroenenberg H. (2009). Indicators for energy security. Energy Policy.

[3] Ahiduzzaman M.D., Islam A.K.M.S. (2011). Greenhouse gas emission and renewable energy sources for sustainable development in Bangladesh. Renew. Sust. Energ. Rev..

[4] Saito K., Irie M., Zaitsu S., Sakai H., Hayashi H., Tanioka A. (2012). Power generation withsalinity gradient by pressure retarded osmosis using concentrated brine from SWROsystem and treated sewage as pure water. Desalin. Water Treat..

[5] Post J.W., Veerman J., Hamelers H.-V.M., Euverink G.-J.W., Metz S.J., Nymeijerc K., Buisman C.J.N. (2007). Salinity-gradient power: Evaluation of pressure- retared osmosis and reverse electrodialysis. J. Membr. Sci..

[6] Avci A.H., Messana D.A., Santoro S., Tufa R.A., Curcio E., Profio G.D., Fontananova E. (2020). Energy harvesting from brines by reverse elecrodialylsis using Nafion membranes. Membranes.

[7] Tufa R.A., Curcio E., Brauns E., Van Baak W., Fontananova E., Di Profio G. (2015). Membrane Distillation and Reverse Electrodialysis for Near-Zero Liquid Discharge and low energy seawater desalination. J. Memb. Sci..

[8] Tufa R.A., Noviello Y., Di Profio G., Macedonio F., Ali A., Drioli E., Fontananova E., Bouzek K., Curcio E. (2019). Integrated membrane distillation-reverse electrodialysis system for energy-ecient seawater desalination. Appl. Energy.

[9] Mei Y., Tang C.Y. (2018). Resent developments and future perspectives of reverse electrodialysis technology: A review. Desalination.

[10] Long R., Li B., Liu Z., Liu W. (2017). Hybrid membrane distillation-reverse electrodialysis electricity generation system to harvest low-grade thermal energy. J. Memb. Sci..

[11] Helfer F., Lemckert C., Anissimov Y.G. (2014). Osmotic power with pressure retarded osmosis: Theory, performance and trends—A review. J. Membr. Sci..

[12] Kim J., Jeong K., Park M.J., Shon H.K., Kim J.H. (2015). Recent advances in osmotic energy generation via pressure-retarded osmosis (PRO): A review. Energies.

[13] Linares R.V., Li Z., Sarp S., Bucs S.S., Amy G., Vrouwenvelder J.S. (2014). Forward osmosis niches in seawater desalination and wastewater reuse. Water Res..

[14] Tedesco M., Scalici C., Vaccari D., Cipollina A., Tamburini A., Micale G. (2016). Performance of the first reverse electrodialysis pilot plant for power production from saline waters and concentrated brines. J. Membr. Sci..

[15] Tedesco M., Cipollina A., Tamburini A., Micale G. (2017). Towards 1 kW power production in a reverse electrodialysis pilot plant with saline waters and concentrated brines. J. Membr. Sci..

[16] Nam J.-Y., Hwang K.-S., Kim H.-C., Jeong H., Kim H., Jwa E., Yang S.C., Choi J., Kim C.-S., Han J.-H. (2019). Assessing the behavior of the feed-water constituents of a pilot-scale 1000-cell-pair reverse electrodialysis with seawater and municipal wastewater effluent. Water Res..

[17] Mehdizadeh S., Kakihana Y., Abo T., Yuan Q., Higa M. (2021). Power generation performance of a pilot-scale reverese elctrodialysis using monovalent selective ion-exchange membranes. Membranes.

[18] Yasukawa M., Suzuki T., Higa M. (2018). Salinity Gradient Processes: Thermodynamics, Application, and Future Prospects (Chapter 1). Membrane-Based Salinity Gradient Processes for Water Treatment and Power Generation.

[19] Yip N.Y., Elimelech M. (2012). Thermodynamic and energy efficiency analysis of power generation from natural salinity gradients by pressure retarded osmosi. Environ. Sci. Technol..

[20] Kim Y.C., Elimelech M. (2012). Adverse impact of feed channel spacers on the performance of pressure retarded osmosis. Environ. Sci. Technol..

[21] She Q., Jin X., Tang C.Y. (2012). Osmotic power production from salinity gradient resource by pressure retarded osmosis: Effects of operating conditions and reverse solute diffusion. J. Membr. Sci..

[22] Hoover L.A., Schiffman J.D., Elimelech M. (2013). Nanofibers in thin-film composite membrane support layers: Enabling expanded application of forward and pressure retarded osmosis. Desalination.

[23] Han G., Zhang S., Li X., Chung T.-S. (2013). High performance thin film composite pressure retarded osmosis (PRO) membranes for renewable salinity-gradient energy generation. J. Membr. Sci..

[24] Zhang S., Fu F., Chung T.-S. (2013). Substrate modifications and alcohol treatment on thin film composite membranes for osmotic power. Chem. Eng. Sci..

[25] She Q., Hou D., Liu J., Tan K.H., Tang C.Y. (2013). Effect of feed spacer induced membrane deformation on the performance of pressure retarded osmosis (PRO): Implications for PRO process operation. J. Membr. Sci..

[26] Patel R., Chi W.S., Ahn S.H., Park C.H., Lee H.-K., Kim J.H. (2014). Synthesis of poly(vinyl chloride)-g-poly(3-sulfopropyl methacrylate) graft copolymers and their use in pressure retarded osmosis (PRO) membranes. Chem. Eng. J..

[27] Han G., Wang P., Chung T.S. (2013). Highly robust thin-film composite pressure retarded osmosis (PRO) hollow fiber membranes with high power densities for renewable salinity-gradient energy generation. Environ. Sci. Technol..

[28] Loeb S., Van Hessen F., Shahaf D. (1976). Production of energy from concentrated brines by pressure-retarded osmosis: II. Experimental results and projected energy costs. J. Membr. Sci..

[29] Chou S., Wang R., Shi L., She Q., Tang C., Fane A.G. (2012). Thin-film composite hollow fiber membranes for pressure retarded osmosis (PRO) process with high power density. J. Membr. Sci..

[30] Zhang S., Chung T.-S. (2013). Minimizing the instant and accumulative effects of salt permeability to sustain ultrahigh osmotic power density. Environ. Sci. Technol..

[31] Fu F.-J., Sun S.-P., Zhang S., Chung T.-S. (2014). Pressure retarded osmosis dual-layer hollow fiber membranes developed by co-casting method and ammonium persulfate (APS) treatment. J. Membr. Sci..

[32] Zhang S., Sukitpaneenit P., Chung T.-S. (2014). Design of robust hollow fiber membranes with high power density for osmotic energy production. Chem. Eng. J..

[33] Wan C.F., Chung T.-S. (2015). Osmotic power generation by pressure retarded osmosis using seawater brine as the draw solution and wastewater retentate as the feed. J. Membr. Sci..

[34] Straub A.P., Yip N.Y., Elimelech M. (2014). Raising the bar: Increased hydraulic pressure allows unprecedented high power densities in pressure-retarded osmosis. Environ. Sci. Technol. Lett..

[35] Higa M., Shigefuji D., Shibuya M., Izumikawa S., Ikebe Y., Yasukawa M., Endo N., Tanioka A. (2017). Experimental study of a hollow fiber membrane module in pressure-retarded osmosis: Module performance comparison with volumetric-based power outputs. Desalination.

[36] Xu Y., Peng X., Tang C.Y., Fu Q.S., Nie S. (2010). Effect of draw solution concentration and operating conditions on forward osmosis and pressure retarded osmosis performance in a spiral wound module. J. Membr. Sci..

[37] Kim Y.C., Kim Y., Oh D., Lee K.H. (2013). Experimental investigation of a spiral-wound pressure-retarded osmosis membrane module for osmotic power generation. Environ. Sci. Technol..

[38] Sakai H., Ueyama T., Irie M., Matsuyama K., Tanioka A., Saito K., Kumano A. (2016). Energy recovery by PRO in sea water desalination plant. Desalination.

[39] Kurihara M., Hanakawa M. (2013). Mega-ton Water System: Japanese national research and development project on seawater desalination and wastewater reclamation. Desalination.

[40] Kurihara M., Sakai H., Tanioka A., Tomioka H. (2016). Role of pressure-retarded osmosis (PRO) in the mega-ton water project. Desalin. Water Treat..

[41] Hayashi H., Okumura T. (2016). Macro and nano behavior of salt water in pressure retarded osmosis membrane module. Desalination.

[42] Kumano A., Marui K., Terashima Y. (2016). Hollow fiber type PRO module and its characteristics. Desalination.

[43] Yasukawa M., Shigefuji S., Shibuya M., Ikebe Y., Horie R., Higa M. (2018). Effect of DS concentration on the PRO performance using a 5-inch scale cellulose triacetate-based hollow fiber membrane module. Membranes.

[44] Tanaka Y., Yasukawa M., Goda S., Sakurai H., Shibuya M., Takahashi T., Kishimoto M., Higa M., Matsuyama H. (2018). Experimental and simulation studies of two types of 5-inch scale hollow fiber membrane modules for pressure-retarded osmosis. Desalination.

[45] Kishimoto M., Tanaka Y., Yasukawa M., Goda S., Higa M., Matsuyama H. (2019). Optimization of pressure-retarded osmosis with hollow-fiber membrane modules by numerical simulation. Ind. Eng. Chem. Res..

[46] Matsuyama K., Makabe R., Ueyama T., Sakai H., Saito K., Okumura T., Hayashi H., Tanioka A. (2021). Power generation system based on pressure retarded osmosis with a commercially-available hollow fiber PRO membrane module using seawater and freshwater. Desalination.

[47] Achilli A., Cath T.Y., Childress A.E. (2009). Power generation with pressure retarded osmosis: An experimental and theoretical investigation. J. Membr. Sci..

[48] Binger Z.M., Achilli A. (2020). Forward osmosis and puressure retarded osmosis process modeling for integration with seawaer reverse osmosis desalination. Desalination.

[49] Touati K., Tadeo F., Kim J.H., Silva O.A.A., Chae S.H. (2017). Oscar Andres Alvarez Silva, Pressure Retarded Osmosis: Renewable Energy Generation and Recovery.

